# Fibronectin Modulates Cell Adhesion and Signaling to Promote Single Cell Migration of Highly Invasive Oral Squamous Cell Carcinoma

**DOI:** 10.1371/journal.pone.0151338

**Published:** 2016-03-15

**Authors:** Grasieli de Oliveira Ramos, Lisiane Bernardi, Isabel Lauxen, Manoel Sant’Ana Filho, Alan Rick Horwitz, Marcelo Lazzaron Lamers

**Affiliations:** 1 Basic Research Center, Dentistry School, Federal University of Rio Grande of Sul, Porto Alegre, Rio Grande do Sul, Brazil; 2 Hospital de Clínicas de Porto Alegre, Porto Alegre, Rio Grande do Sul, Brazil; 3 Department of Cell Biology, University of Virginia, Charlottesville, Virginia, United States of America; 4 Department of Morphological Sciences, Institute of Basic Health Sciences, Federal University of Rio Grande do Sul, Porto Alegre, Rio Grande do Sul, Brazil; Pennsylvania State Hershey College of Medicine, UNITED STATES

## Abstract

Cell migration is regulated by adhesion to the extracellular matrix (ECM) through integrins and activation of small RhoGTPases, such as RhoA and Rac1, resulting in changes to actomyosin organization. During invasion, epithelial-derived tumor cells switch from laminin-enriched basal membrane to collagen and fibronectin-enriched connective tissue. How this switch affects the tumor migration is still unclear. We tested the hypothesis that ECM dictates the invasiveness of Oral Squamous Cell Carcinoma (OSCC). We analyzed the migratory properties of two OSCC lines, a low invasive cell line with high e-cadherin levels (L^inv^/H^E-cad^) or a highly invasive cell line with low e-cadherin levels (H^inv^/L^E-cad^), plated on different ECM components. Compared to laminin, fibronectin induced non-directional collective migration and decreased RhoA activity in L^inv^/H^E-cad^ OSCC. For H^inv^/L^E-cad^ OSCC, fibronectin increased Rac1 activity and induced smaller adhesions, resulting in a fast single cell migration in both 2D and 3D environments. Consistent with these observations, human OSCC biopsies exhibited similar changes in cell-ECM adhesion distribution at the invasive front of the tumor, where cells encounter fibronectin. Our results indicate that ECM composition might induce a switch from collective to single cell migration according to tumor invasiveness due to changes in cell-ECM adhesion and the resulting signaling pathways that alter actomyosin organization.

## Introduction

Oral squamous cell carcinoma (OSCC) is an epithelial neoplasm found in 80–90% of head and neck cancer [1]. OSCC can occur at several sites of the oral mucosa and is originated from genetically altered keratinocytes arising from exposure to a wide range of mutagenic agents [2]. Histopathologically, OSCC lesions are characterized by the presence of different degrees of squamous differentiation, keratin production, nuclear pleomorphisms, mitotic activity, invasive growth and metastasis. Despite advances in treatment, the OSCC prognosis remains poor with a 5 year survival rate of around 50%. This prognosis has not improved over the past several years due to the development of distant metastasis, local recurrences and new tumors [1, 3, 4].

The ability of tumor cells to invade connective tissue is essential for them to access blood vessels and ultimately promote distant metastasis. Both events, tissue invasion and metastasis, are highly heterogeneous processes [5], requiring tumor cell adaptation to new environments that alter the migratory mode. Depending on the tumor origin, differentiation level, and tumor microenvironment, cancer cells migrate either as collective or single cells [6]. Amoeboid- and mesenchymal-like single cell migration involve the coordinated interaction of structural and signaling molecules that results in polymerization of actin at the leading edge, adhesion to the extracellular matrix (ECM) through integrins, contraction of the cell cortex and detachment of adhesions at the cell rear [7, 8], whereas cluster or strand like collective cell migration involves the single cell migration steps associated with the presence of cell-cell contacts, mainly mediated by cadherin family members [6, 9]. Rho family GTPases orchestrates changes in actomyosin organization that drive these key events in cell migration. For example, Rac1 regulates actin filament nucleation associated with nascent adhesion formation, and RhoA controls cell contractility, actin elongation and adhesion maturation [7, 10]. Changes in RhoGTPase activation levels interfere with the balance between cell-cell and cell-ECM adhesions and likely influences collective vs single cell migration [10–13].

Tumor formation is sensitive to the microenvironment, which varies by the region of the tumor. The tumor microenvironment is characterized by intense angiogenesis, high concentrations of growth factors and inflammatory cytokines, and ECM remodeling [14, 15]. An abrupt adaptation occurs during invasion of epithelial-derived tumors when they move from the basal membrane, a laminin enriched environment, to the connective tissue region, which is rich in collagen and fibronectin [16, 17]. Oral squamous cell carcinoma biopsies exhibit decreased laminin content and increased fibronectin, depending on the aggressiveness and the location of the tumor [18, 19]. It is likely that the characteristics of the tumor microenvironment, such as the composition of the extracellular matrix, influence metastatic and invasive behavior due to biochemical or physical activation of migration-related proteins and signaling pathways.

In this study, we report that the change from a laminin- to a fibronectin-rich environment has a differential effect on the migration properties of OSCCs. In high invasive and low E-cadherin expressing OSCC cells (H^inv^/L^E-cad^), fibronectin induced a fast single cell migration phenotype that is associated with increased Rac1 activation levels and small cell-ECM adhesions; in low invasive and high E-cadherin OSCC cells (L^inv^/H^E-cad^), fibronectin produces a collective, non-directional migration, with high RhoA activity and altered cell-ECM adhesion. Consistent with these results, human OSCC biopsies also demonstrated changes in cell-ECM and cell-cell adhesion according to the tumor region. Together, these data show that the composition of the extracellular matrix differentially affects cell-ECM adhesion, cell migration signaling pathways and the migratory output of OSCC cells and that these effects vary according to the differentiation level of the tumor.

## Material and Methods

### Human Biopsies and OSCC Cell Culture

The experimental design and the informed consent procedures were approved by the Ethical Committee of Federal University of Rio Grande do Sul—Brazil and of Hospital de Clínicas de Porto Alegre—Brazil (CAE#06397313.7.0000.5347) and all patients in this study provided written informed consent. Patients (n = 10) with oral lesions were interviewed and submitted to surgery; OSCC diagnosis was confirmed histopathologically by a pathologist and fragments from regions corresponding to the center of the tumor and the carcinoma edge tissue, named as tumor adjacent epithelia (TAE) were collected. OSCC cell lines were obtained from the Tissue Culture Facility at School of Medicine of University of Virginia and checked for mycoplasma by this facility. Cal27 cells (ATCC^®^ CRL-2095^™^) were cultivated in DMEM high glucose (Gibco) supplemented with 10% Fetal Bovine Serum (FBS) (Gibco) while SCC25 cells (ATCC^®^ CRL-1628^™^) in DMEM/F12 with 15mM HEPES and 0.5mM sodium pyruvate (Gibco) supplemented with FBS 10% and hydrocortisone (400ng/ml, Sigma), and cells were maintained in incubator (37°C, 5% CO_2_). Cal27 cells are considered low invasive OSCC cells [20] with high E-cadherin levels (L^inv^/H^E-cad^), while SCC25 cells are highly invasive with low E-cadherin levels (H^inv^/L^E-cad^). Spheroids were performed plating 5x10^4^ cells in a 96 wells dish covered with 1.5% agarose and, after 3 days, spheroids were gently collected and used for experiments. For Total Internal Reflectance Fluorescence (TIRF) microscopy, cell lines (1x10^6^) were nucleofected 24h before the experiment with 0.2μg Paxillin-GFP plasmid [21], using Amaxa Nucleofection System (Lonza).

### Experimental Conditions

Unless stated otherwise, all reagents were purchased from Sigma Aldrich. For 2D imaging experiments, cells were trypsinized, washed and plated in glass-bottomed dishes covered with fibronectin (2μg/ml), laminin (poly-l-lysine (1mg/ml) + laminin (2μg/ml)) or Matrigel^®^ (50μl/cm^2^, BD Bioscience) in the presence of CCM1 media (Hyclone, Thermo Scientific). For 3D imaging experiments, it was used collagen (1.2mg/ml, rat tail collagen) matrices assembled according to the manufacturer (Gibco) in the presence/absence of fibronectin (10μg/ml) or laminin (10μg/ml). For each condition, a thin layer of the respective collagen matrix was initially plated at the surface of the glass-bottomed dishes. After polymerization, 3x10^4^ cells or spheroids were embedded in a new collagen matrix and, after 3h, imaged using CCM1 media. To ensure that cells were in the 3D matrix, it was verified the lower and the upper focus with detectable cells and it was always selected cells for imaging at an intermediate focus position.

### Immunoblots

Antibodies were purchased from Cell Signaling (E-cadherin, N-cadherin, Integrins α4, α5, αv, β1, β3), BD-Transduction (Paxillin, FAK) and Sigma (β-Tubulin, Vinculin). Cells (1x10^6^) were trypsinized, washed and lysed in RIPA Buffer (25mM Tris-HCL pH 7.6, 150mM NaCl, 1% NP-40, 1% sodium deoxycholate, 0.1% SDS) containing protease and phosphatase inhibitors cocktails. Cell lysates (20μg) were separated in 4–20% SDS Gels (Biorad) and proteins transferred to PVDF membranes, blocked (4% BSA) and immunoassayed for E-cadherin, FAK, Paxilllin, β-Tubulin, Vinculin or integrins (α4, α5, αv, β1, β3) using Pierce ECL Western Blotting Substrate (Thermo Scientific). Densitometry of the bands was performed using ImageJ software (http://rsb.info.nih.gov/ij), and values for each protein were normalized to the loading control.

### Immunofluorescence

For tumor staining, human biopsies were fixed immediately after collection (4% formaldehyde, 4h, 4°C), cryoprotected with increasing sucrose concentrations (10–30%, 4°C), embedded in OCT compound, frozen (-20°C), cut using cryostat and seven μm-thick slices were collected in gelatin-covered slides. For cell lines staining, L^inv^/H^E-cad^ and H^inv^/L^E-cad^ were plated in coverslips covered with fibronectin (2μg/ml) or poly-l-lysine (1mg/ml) + laminin (2μg/ml) in the presence of CCM1 media. After 3h, cells were washed (PBS) and fixed (formaldehyde, 4%, 10min, RT). Fixed cells or human biopsies were permeabilized (Triton X-100 0.3%, RT 10 min), blocked (10% normal goat serum, RT, 1h), incubated with antibodies for E-cadherin, FAK, Paxillin, Vinculin or Fibronectin (ON, 4°C), washed (PBS) and incubated (2h, RT) with the corresponding secondary antibodies containing Alexa488 dye (Molecular Probes, Oregon, USA). Actin filaments were stained with phalloidin toxin conjugated to rhodamine (Molecular Probes, Oregon, USA) for 2h (RT). Samples were washed (PBS) and mounted with antifade medium (Vectashield, VectorLab, Burlingame, CA). Images were obtained in confocal microscope (Olympus Fluoview 1000, Tokyo, Japan) with a 63x objective (UPlanSApo x63, 1.20 NA, oil immersion objective) using FV-1000 ASW Fluoview software (Olympus, Tokyo, Japan). Alexa488 was excited with the 488nm laser line of an Argon ion laser (Melles Griot, Albuquerque, NM), while rhodamine with the 543nm laser line of a Helium-Neon laser (Melles Griot, Albuquerque, NM). Z-stacks were obtained from cells (0.1μm step size) and biopsy slices (0.5μm step size) with or without digital zoom (3x for cell lines; 5x for biopsies). In order to analyze the whole adhesion and avoid image background, 3 confocal-obtained slices were merged using the “Z-stack/maximum projection” tool from the ImageJ software. This new merged image corresponds to an equivalent 0.3μm or 1.5μm thick slice of the cell lines or the biopsy samples, respectively. Besides brightness/contrast corrections, no further image editing was performed and figures were prepared using Adobe Photoshop^®^ 7 software.

### RhoGTPase Activity

For analysis of RhoGTPase activation, pull down assays [22] were performed. L^inv^/H^E-cad^ and H^inv^/L^E-cad^ cells were plated in plastic dishes covered with fibronectin (2μg/ml) or poly-l-lysine (1mg/ml) + laminin (2μg/ml) in the presence of CCM1 media. After 3h, cells were washed (PBS), harvested, lysed with CRIBs buffer in the presence of protease and phosphatase inhibitors and incubated in the presence of GST-PAK-CRIB (Rac1) or GST-RBD-CRIB (RhoA) beads. After washing, samples were prepared for SDS-PAGE and submitted to immunoblotting for Rac1 (BD Bioscience) or RhoA (Santa Cruz Biotechnologies). Densitometry of the bands was performed using ImageJ software.

### FRET Imaging and Analysis

L^inv^/H^E-cad^ and H^inv^/L^E-cad^ OSCC cells were nucleofected with Raichu-Rac1-WT or Raichu-Rac1-V12 plasmids for Rac1 activity and Raichu–RhoA-WT or Raichu-RhoA-Q63L plasmids [23] for RhoA activity (0.5μg/10^6^ cells) and 24h later were trypsinized and plated in plastic dishes covered with fibronectin (2μg/ml) or poly-l-lysine (1mg/ml) + laminin (2μg/ml) in the presence of CCM1 media. Cells were washed (PBS), fixed in formaldehyde 4% and sacarose 4% (10 min, RT), washed (PBS) and analyzed by confocal microscopy with 2x digital zoom. Donor probe was excited with the 458nm laser line of an Argon ion laser (Melles Griot, Albuquerque, NM). Images were analyzed by Matlab^®^ software (MathWorks, Natick, MA) using the Biosensor Processing software 2.1 [24]. The mean intensity values from FRET-ratio TIFF images were obtained on ImageJ software. Using ImageJ software, selected images were adjusted for the same levels of brightness/contrast and a 0.5 pixel-wide Gausian filter was applied.

### Migration and Adhesion Dynamics Assays

Imaging acquisition and analysis for migration assays were performed as previously described [25]. For phase microscopy movies, images were captured at 10min intervals using a Nikon TE300 microscope (10x 0.25 NA CFI Achro DL106 Nikon objective) with a charge coupled device camera (Orca II, Hamamatsu Photonics) using Metamorph software (Molecular Devices). For TIRF microscopy, images were taken at 3s intervals using an Olympus IX70 inverted microscope (63x 1.45 NA oil Olympus PlanAplo 660 TIRFM objective) fitted with a Ludl modular automation controller (Ludl Electronic Products) with a charge-couple device camera (Retiga Exi, Qimaging) and controlled by Metamorph software. GFP was excited with the 488nm laser line of an Argon laser (Melles Griot) and a dichroic mirror (HQ485/30) and an emission filter (HQ525/50) were used. All images and movies were analyzed using ImageJ software and panels mounted using Adobe Photoshop^®^ 7 software. For analysis of migration parameters, it was performed at least 4 independent experiments (phase contrast microscopy movies) and the nucleus of each migratory cell was tracked using the “manual tracking” plug-in on ImageJ. It was considered as migratory cell only cells that migrated for at least 6h. In case of migratory cells that underwent mitosis, the tracking process was ended 1h before cytokinesis. To determine migration speed, it was performed the ratio between the total distance traveled (distance) and the number of slices (time) that cell migrated. To analyze the cell trajectory and persistence of migration, the X and Y coordinates obtained during the tracking of the nucleus of the migratory cell in each slice were normalized to start at a virtual X = 0 and Y = 0 position and the variation on the position was plotted in a polar plot graph [25]. For analysis of adhesion properties, it was used H^inv^/L^E-cad^ cells expressing paxillin-GFP from at least 4 independent experiments (TIRF microscopy) for each experimental group. Adhesion length and area was determined by measuring, respectively, the long axis or the area of each adhesion that assembled during the movie. The percentage of total adhesion area in each newly formed protrusion was measured by the ratio of the sum of the area of all adhesions that assembled in the protrusion by the total area of the protrusion. The adhesion assembly speed was measured using the “kymograph” plug-in on ImageJ. For each adhesion, a line (1 pixel-wide) was drawn in the long axis of the adhesion and the X (distance) and Y (time) coordinates originated by the kymograph were used to measure the speed of adhesion assembly. All data were calculated using Microsoft Excel^®^ (Microsoft Corporation) and SPSS 21 software (Statistical Package for the Social Science, IBM).

### Statistical Analysis

Student t test or One-way analysis of variance (ANOVA) followed by Tukey’s post-test were employed, using SPSS 21 software and differences were considered significant when p<0.05.

## Results

### Fibronectin Induces Fast Single Cell Migration of Highly Invasive OSCCs Cells

**Fig 1:**
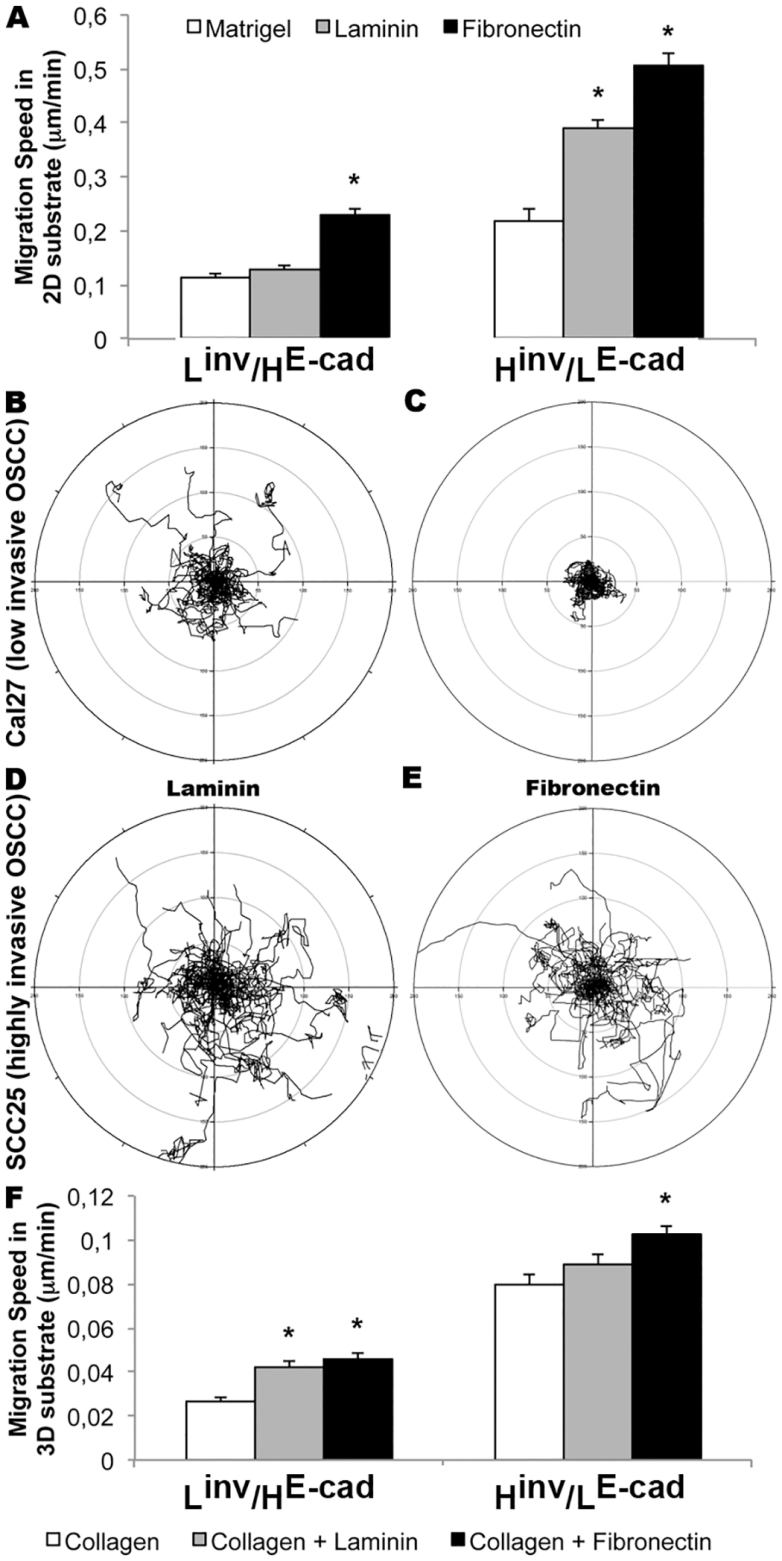
Fibronectin induces faster migration speed in 2D and 3D substrates. (A) Effects of different 2D substrates on migration speed (24h) of L^inv^/H^E-cad^ (Cal27) or H^inv^/L^E-cad^ (SCC25) OSCC cell lines (n = 3); (B-E) Cell migration trajectory of L^inv^/H^E-cad^ (B-C) or H^inv^/L^E-cad^ (D-E) cells plated on laminin (B and D) or fibronectin (C and E); (F) Effects of different 3D substrates on migration speed of L^inv^/H^E-cad^ and H^inv^/L^E-cad^ cell lines (n = 3). Results are expressed as mean ± SEM. (*) p<0.05 according to One-way analysis of variance (ANOVA) followed by Tukey’s post-test.

Since the extracellular matrix composition can influence the migratory properties of various cell types, L^inv^/H^E-cad^ (Cal27) or H^inv^/L^E-cad^ (SCC25) oral squamous cell carcinoma cell lines [20] were plated on 2D- Matrigel^®^ (50μl/cm^2^), laminin (2μg/ml) or fibronectin (2μg/ml)-coated glass bottomed dishes and imaged for 24h. We tracked the migration velocity of individual as well as group of cells. Matrigel and laminin were used to mimic the laminin-rich basement membrane that supports cells in an epithelial sheet, whereas fibronectin was used to challenge the cells with the connective tissue matrix encountered when cells metastasize. On Matrigel, both cell lines migrated collectively (S1 Movie), while on laminin, both cell types exhibited collective as well as single cell migration (S2 and S3 Movies). While L^inv^/H^E-cad^ cells showed no changes in migration speed, H^inv^/L^E-cad^ cells exhibited a ~40% increase in migration speed on laminin when compared to Matrigel (Fig 1A). When cells were plated on fibronectin, both cell types migrated faster than on laminin, and exhibited pronounced changes in directionality. Both OSCC lines showed a ~40% increase in migration speed; but L^inv^/H^E-cad^ cells migrated collectively in circles (S2 Movie), whereas H^inv^/L^E-cad^ cells migrated as single cells with persistent directionality (Fig 1B, S3 Movie).

To complement the observations in a 2D environment, L^inv^/H^E-cad^ or H^inv^/L^E-cad^ OSCC cells were plated in a 3D matrix, containing collagen (1.2mg/ml), collagen + laminin (1.2mg/ml+10μg/ml) or collagen + fibronectin (1.2mg/ml+10μg/ml), and imaged for 24h. When compared to a 3D collagen only gel, L^inv^/H^E-cad^ cells showed a ~50% increase in migration speed when plated in 3D collagen gel containing laminin or fibronectin (Fig 1F, S4 Movie) with a slight increase in directional persistence when plated on collagen+laminin. H^inv^/L^E-cad^ tumor cells showed no changes in migration speed when plated in a collagen+laminin 3D environment, but were able to invade the collagen gel when plated in collagen+fibronectin matrices (Fig 1F, S5 Movie). Both cells migrated poorly when plated in a 3D matrix containing only collagen.

Since OSCC biopsies exhibit tumor islands inside the connective tissue, we developed spheroids from both cell lines, plated them in a collagen (1.2mg/ml) or a collagen+fibronectin (1.2mg/ml+2μg/ml) 3D environment, and imaged for 36h. S6 movie shows that small or big spheroids derived from L^inv^/H^E-cad^ OSCCs proliferated, but showed little migratory activity. However, spheroids of the H^inv^/L^E-cad^ OSCC cells showed cells that migrated out of the spheroid and invaded the surrounding tissue only when plated in a collagen+fibronectin 3D environment.

To summarize, these results in 2D and 3D matrices show that L^inv^/H^E-cad^ OSCC cells migrate more directionally when plated using conditions similar to the epithelial and blood vessel basal lamina; whereas H^inv^/L^E-cad^ tumor cells switch from a collective to a faster single cell migration when transitioning from a laminin to a fibronectin rich connective tissue-like environment.

### OSCCs Extracellular Matrix-Derived Migration Properties Are Associated with Changes in RhoGTPase Signaling

**Fig 2:**
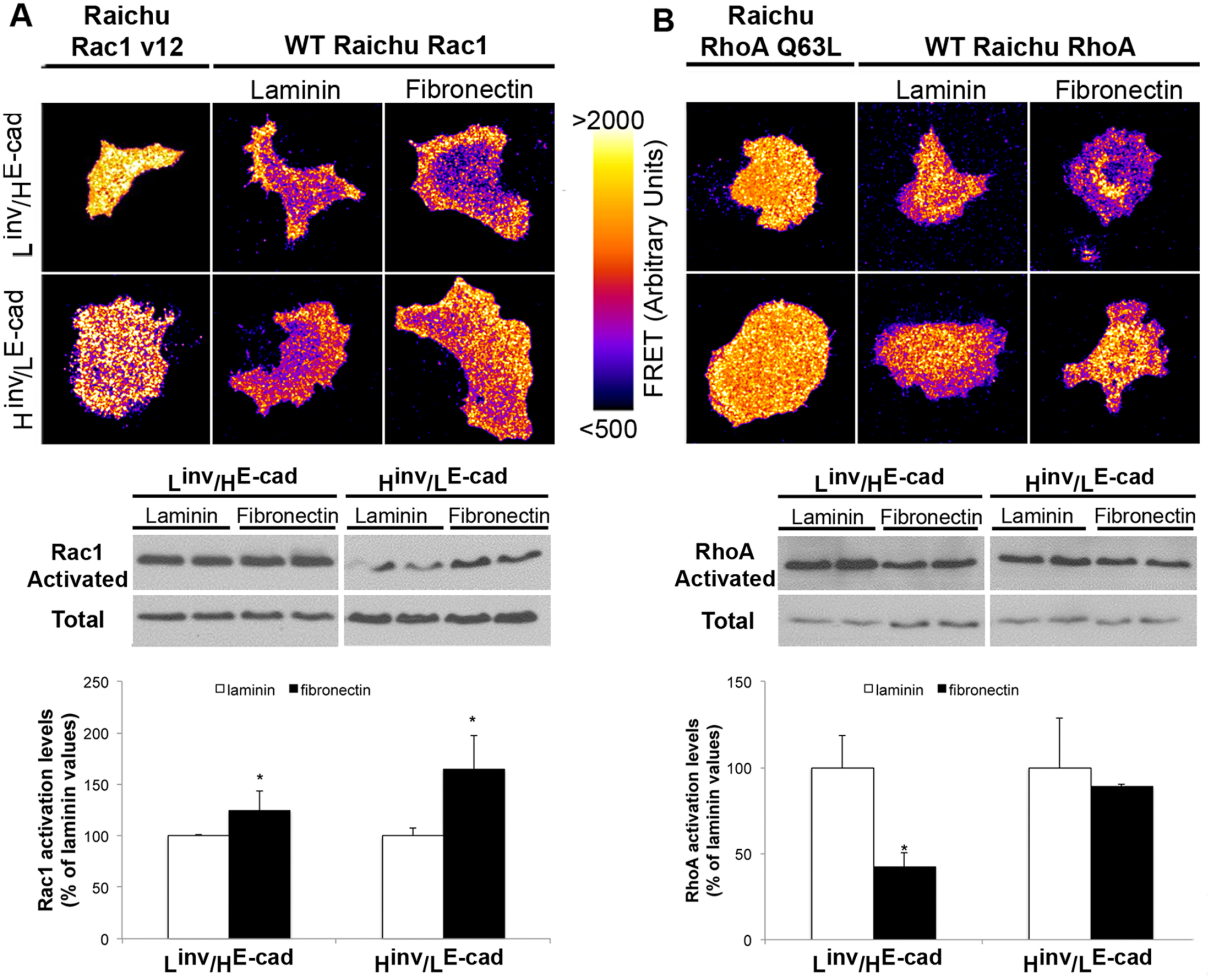
RhoGTPase activation varies according to extracellular matrix composition and tumor differentiation levels. FRET analysis and pull down assay for Rac1 (A) and RhoA (B) of L^inv^/H^E-cad^ (Cal27) or H^inv^/L^E-cad^ (SCC25) OSCC plated in laminin (2μg/ml) or fibronectin (2μg/ml). Raichu-Rac1-V12 and Raichu-RhoA-Q63L represents the constitutively activated isoform. Results are expressed as mean ± SD. (*) p<0.05, n = 4.

A differential activation of RhoGTPase signaling is a likely mechanism for the ECM-derived differences in cell migration observed in the L^inv^/H^E-cad^ and H^inv^/L^E-cad^ OSCC cell lines. To address this, we analyzed the Rac1 and RhoA activation levels by pull down and FRET assay of cells plated on either laminin (2μg/ml) or fibronectin (2μg/ml) coated dishes. Consistent with the increased migration speed observed for both cell types on fibronectin, fibronectin increased Rac1 activation levels when compared to cells plated on laminin, which was accompanied by a FRET signal mainly at the cell borders (Fig 2A); this effect was slightly, but consistently, more pronounced in the H^inv^/L^E-cad^ tumor cells. In contrast, RhoA activity was observed mainly at the cell body and showed a decrease in the L^inv^/H^E-cad^ cells plated on fibronectin, but was unaltered in the H^inv^/L^E-cad^ cell line (Fig 2B). This decreased RhoA activity may reflect the fact that L^inv^/H^E-cad^ cells migrate collectively on fibronectin, whereas the H^inv^/L^E-cad^ cells migrate as single cells, where RhoA is necessary for formation of the contractile cell rear underlying persistent directional migration [26]. These data indicate that the effects of ECM constitution on tumor invasion process involve a differential activation of RhoGTPases that varies according to the aggressiveness and differentiation level of the tumor cells.

### Extracellular-Matrix Composition Interferes with Tumor Cell Adhesion Properties

**Fig 3:**
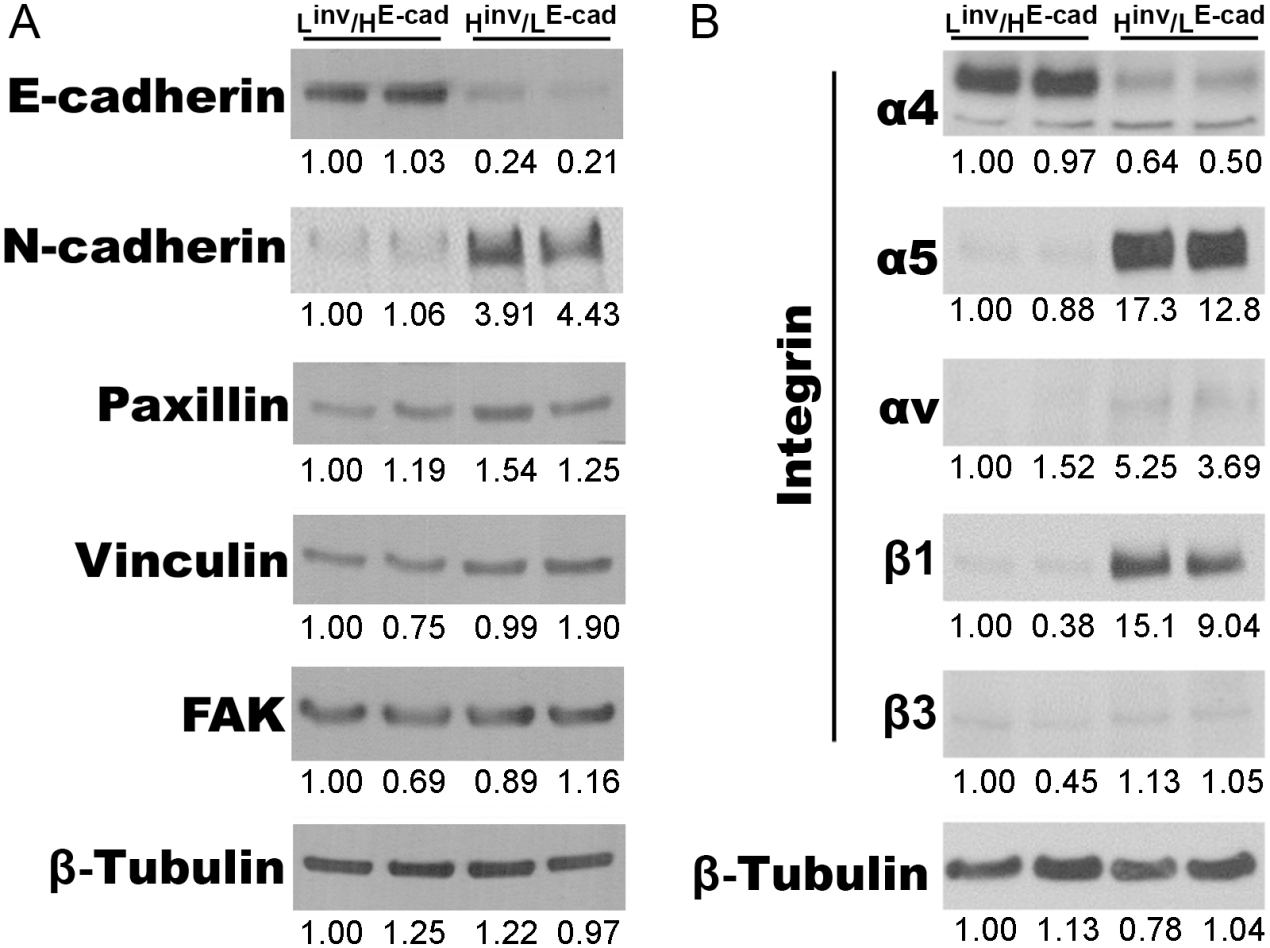
Decreased cell-cell and increased cell-ECM adhesion proteins characterize invasive OSCC. Representative western blotting images of cell-cell (E-cadherin, N-cadherin), cell-ECM (paxillin, vinculin and FAK) and integrins (α4, α5, αv, β1 and β3) from L^inv^/H^E-cad^ (Cal27) or H^inv^/L^E-cad^ (SCC25) OSCC total cell lysates. Densitometry values for each protein were normalized to the loading control (β-Tubulin).

Since L^inv^/H^E-cad^ OSCCs migrated collectively, whereas H^inv^/L^E-cad^ OSCCs migrated as single cells, we asked whether these effects correlated with the ratio of cell-cell versus cell-ECM adhesions. We hypothesized that L^inv^/H^E-cad^ cell line, which exhibit collective cell migration, would exhibit increased cell-cell adhesion markers, notably cadherin, whereas highly invasive single cells would most likely favor cell-ECM adhesions. Consistent with this hypothesis, by western blotting, we observed that both cell lines expressed integrins for fibronectin, but H^inv^/L^E-cad^ OSCCs presented an increase in the expression levels of α5 and β1 (Fig 3B) when compared to L^inv^/H^E-cad^ OSCCs. Also, H^inv^/L^E-cad^ OSCCs show a slightly increased expression of cell-ECM adhesion related proteins, including the nascent adhesion marker paxillin, the mechanosensing modulator vinculin and the adhesion signaling marker focal adhesion kinase (FAK). Additionally, H^inv^/L^E-cad^ OSCCs gained expression of N-Cadherin, a marker of the epithelial to mesenchymal transition (Fig 3A). These data suggest that epithelial-derived tumor cells show a differential expression of cell-cell and cell-ECM adhesion markers according to the differentiation levels.

**Fig 4:**
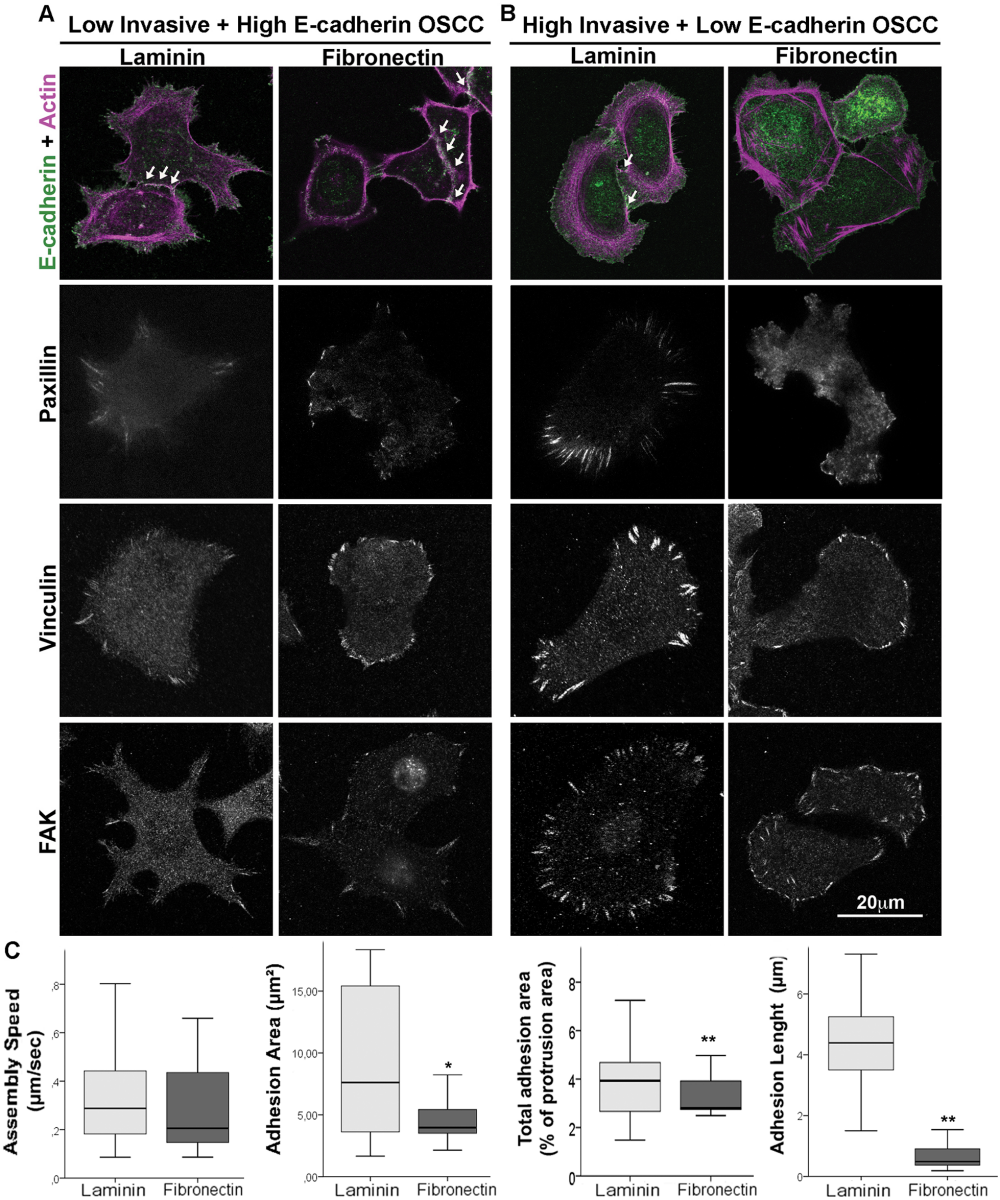
Fibronectin induces smaller adhesion on low E-cadherin expression OSCC cell line. L^inv^/H^E-cad^ (A) or H^inv^/L^E-cad^ (B) invasive OSCC were plated on laminin or fibronectin, fixed and stained for E-cadherin and actin, paxillin, vinculin and FAK. White arrows indicate the signal of E-cadherin between cells. Scale bar = 20μm. Data regarding adhesion properties (C) were obtained using Total Internal Reflectance Fluorescent microscopy analysis of H^inv^/L^E-cad^ OSCC cells expressing paxillin-GFP and plated on laminin (light gray) or fibronectin (dark gray). The data shows the assembly speed (μm/sec), adhesion area (μm^2^), total adhesion area (as % of total protrusion area) and adhesion length (μm). Results are expressed as mean ± SEM. (*) p = 0.05; (**) p < 0.01, according to Student T—test.

In order to analyze the effects of ECM on cellular distribution of adhesion markers, we observed by immunofluorescence that L^inv^/H^E-cad^ OSCCs showed a localization of E-cadherin preferentially at the cell-cell contacts on both fibronectin and laminin (Fig 4A, S1 Fig) while the ECM adhesion markers, paxillin, vinculin and FAK, all localized primarily to large, elongated adhesions, as is often observed in slow migratory cells. By contrast, H^inv^/L^E-cad^ OSCC cells displayed mainly cytoplasmic E-cadherin with only weak staining at cell-cell contacts in both ECM environments (Fig 4B). On laminin, paxillin, vinculin and FAK localized to elongated adhesions similar to L^inv^/H^E-cad^ OSCCs. On fibronectin, however, these adhesion markers preferentially redistributed from large adhesions to small adhesions at the cell border, consistent with the observed increase in Rac activity and the faster migration speed. To confirm these effects of ECM composition on the adhesion of H^inv^/L^E-cad^ OSCCs, we performed live cell imaging (TIRF microscopy) of cells expressing the nascent adhesion marker paxillin-GFP in order to analyze adhesions properties during protrusion (Fig 4C and S7 movie). While there was no difference in adhesion assembly speed when H^inv^/L^E-cad^ OSCCs were plated on fibronectin or laminin, fibronectin decreased adhesion length by ~80% (p ≤ 0.001, n = 43 adhesions, Student T test), and similarly decreased both individual adhesion area by ~50% (p ≤ 0.05, n = 221 adhesions, Student T test) as well as total adhesion area relative to protrusion area by 30% (p ≤ 0.01, n = 17 protrusions, Student T test). These data indicate that the switch from laminin to fibronectin induces smaller adhesions on H^inv^/L^E-cad^ OSCCs cells, which is consistent with the phenotype of highly migratory cells. Thus, the increased persistent migration of H^inv^/L^E-cad^ OSCCs on fibronectin at least in part reflects a preference for cell-ECM adhesion, particularly nascent signaling adhesions, rather than cell-cell adhesions.

### Human Oral Squamous Cell Carcinoma Biopsies Show Increased Levels of Cell-ECM Adhesion Proteins

**Fig 5:**
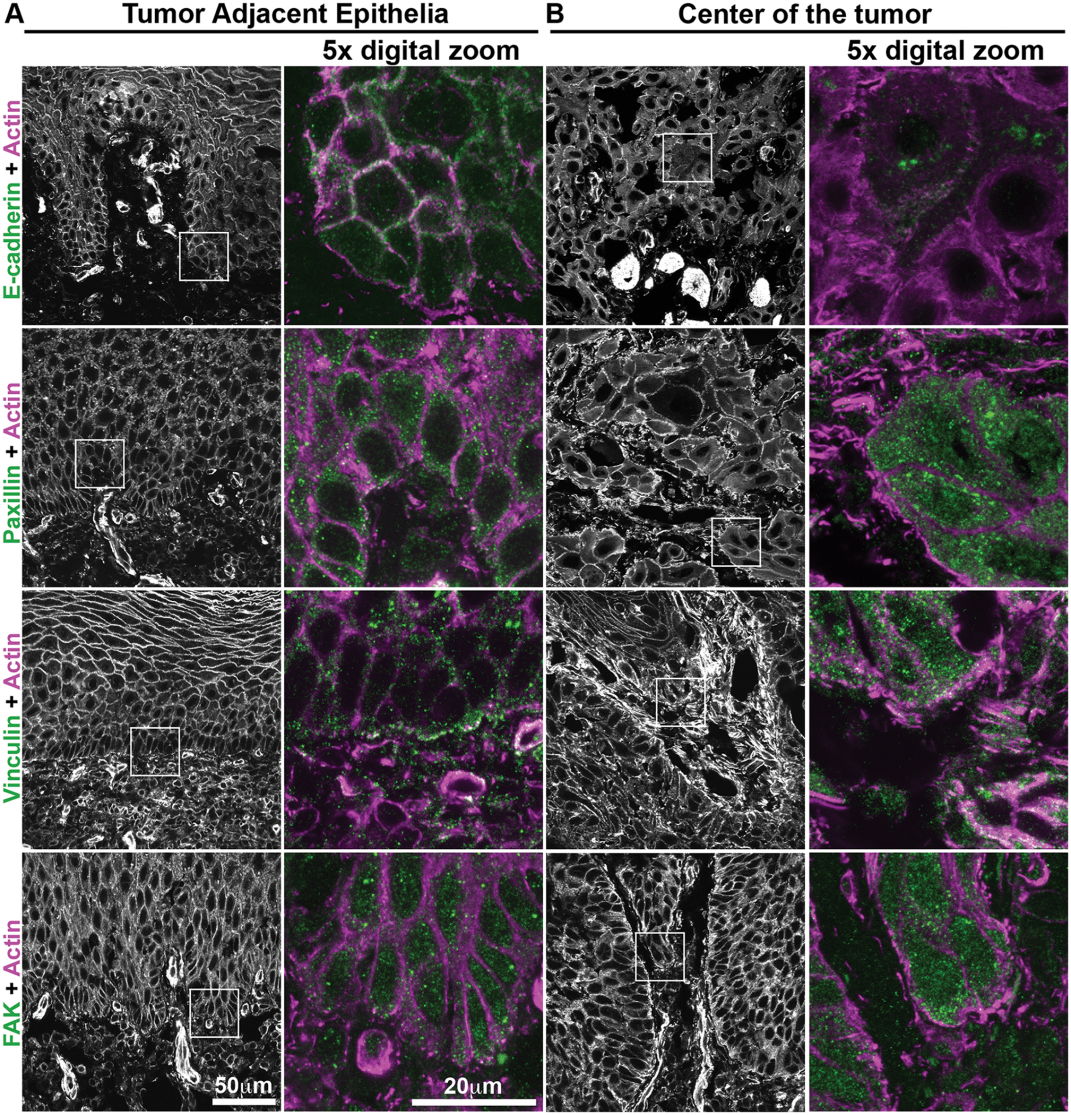
Human oral squamous cell carcinoma biopsies show differential distribution of adhesion proteins between center of the tumor cells and tumor-adjacent epithelia. Regions of biopsies corresponding to the epithelia adjacent to the tumor (A) and from the center of the tumor (B) were submitted to immunostaining for E-cadherin, paxillin, vinculin or FAK (green) and actin staining (magenta). Inserts demonstrated in actin staining, were digitally magnified (5x) to show intracellular localization. Representative images from different patients (n = 10), scale bar = 50μm or 20μm.

Since ECM-associated changes on OSCC invasiveness are likely driven by changes in adhesion, we analyzed the adhesion proteins on biopsies from 10 patients. The sections were stained for fibronectin, E-cadherin, paxillin, vinculin, and FAK and analyzed using confocal microscopy. We compared regions from Tumor Adjacent Epithelia (TAE) (Fig 5, columns 1 and 2; S2 and S3 Figs) with regions at the center of the tumor (Fig 5, columns 3 and 4; S2 and S3 Figs). Fibronectin signal was observed in the connective tissue in both regions (S3 Fig). E-cadherin labeling in TAE cells was strongest at cell-cell contacts and co-localized with actin; while the center of the tumor cells showed a weak signal mainly within the cytoplasm, suggesting protein degradation and/or mislocalization from junctional regions. In TAE regions, paxillin and FAK stained weakly in puncta through the cytoplasm of the epithelia basal layer and weakly co-localized with actin; while vinculin was present mainly at the basal membrane. In the center of tumor, cells that appeared to have detached from the tumor island showed an increase in the staining of proteins related to cell-ECM adhesion, with paxillin showing increased labeling at the cell border, close to the ECM, while vinculin and FAK were observed at regions of membrane extensions of cells at the periphery of the tumor island, with some co-localization with actin. Thus, human biopsies exhibit changes in the distribution of cell-ECM adhesion proteins, particularly at the invasive front of the tumor, that correspond with changes observed in cell-ECM adhesion of H^inv^/L^E-cad^ OSCC plated on fibronectin.

## Discussion

Clinical failures in cancer therapies are due in part to the plasticity of tumor cells to a changing microenvironment [14, 15]. For example, tumor cells physically and biochemically alter extracellular matrix organization [27], which appears to impact several aspects of the epithelial-to-mesenchymal transition (EMT) and cell survival [28, 29]. Furthermore, the migration of epithelial-derived tumors can vary from collective to single cell migration, reflecting changes in tumor cell-cell adhesion and the structure of the tissue that the cells are invading [6, 30, 31]. In glioblastomas, for example, targeted depletion of fibronectin modifies collective cell migration, making cancer cells sensitive to ionizing radiation [32].

We have demonstrated that 2D substrates resembling the epithelial basal membrane induce collective cell migration in OSCC cells independently of the levels of the cell-cell adhesion protein E-cadherin. In contrast, fibronectin-enriched 2D or 3D environments induced single cell, mesenchymal-like cell migration but specifically in the H^inv^/L^E-cad^ OSCCs. Gaggioli et al (2007) [17] demonstrated, in a 3D collagen environment, that squamous cell carcinoma cells showed invasive behavior due to the fibroblast-mediated proteolytic ECM remodeling and fibronectin deposition. Fibronectin also developmentally regulates migration during embryogenesis and determines cell fate [33]. Similarly, fibronectin is overexpressed at the invasive zone of OSCC biopsies [18, 19], where it likely contributes to the abnormal invasive behavior of poorly differentiated cells [34].

Here we sought to determine how the ECM composition of different tumor regions affects cell migration and the signaling mechanisms underlying these different migratory properties. Cell migration is regulated mainly by RhoGTPases, where Rac1 stimulates actin polymerization and nascent adhesion formation while RhoA controls cell contractility and adhesion maturation [7, 10]. During tumor invasion, the balance of RhoGTPase activation is disrupted. Rac1 activation results in a loss of cell junctions and polarity and increased cell motility [11, 35]. Chen et al 2013 [36] demonstrated that mammary epithelial cells undergo EMT when plated on fibronectin, through a mechanism that involves Rac1b activation, while laminin suppresses EMT. Consistent with this observation, increased Rac1 activity drives mesenchymal-like, single cell migration of other cancer cells undergoing EMT [6, 12]. Yap, et al 2009 [37] demonstrated that different OSCC cell lines show increased Rac1 activation when plated on fibronectin, indicating that the microenvironment can influence the tumor invasive behavior through the modulation of cell migration-related signaling pathways. In this study, when compared to laminin, we showed that fibronectin induced an increase in Rac1 activation and a rapid single cell migration phenotype in H^inv^/L^E-cad^ OSCCs. Interestingly, while L^inv^/H^E-cad^ OSCCs also exhibited increased Rac1 activation on fibronectin, they had lower RhoA activation. This decreased RhoA activation may account for the decreased directionality of L^inv^/H^E-cad^ OSCCs, which tended to migrate collectively in circles, since RhoA-mediated myosin activation promotes persistent directional migration [26]. Thus, differential RhoGTPase activity might contribute to migration speed and persistence, as well as collective versus single cell migration on different substrates.

In addition to differential RhoGTPase expression, we observed changes in cell-ECM adhesions on different substrates, with fibronectin favoring the formation of small nascent adhesions in H^inv^/L^E-cad^ OSCCs [38–40]. A possible explanation for the selective effect of fibronectin in our study is the higher expression of fibronectin-related integrins observed in H^inv^/L^E-cad^ OSCCs when compared to L^inv^/H^E-cad^ cells. Integrins are a family of transmembrane proteins that mediate the binding of ECM proteins with intracellular proteins, resulting in biochemical and mechanical signaling pathways that influence several steps on tumor progression [41–44]. Consistent with our results, Chen et al 2012 [45] showed that laminin induced elongated and fluxing adhesions in CHO.K1 cells, while fibronectin induced smaller and more dynamic adhesions. Similarly, we demonstrated that both, L^inv^/H^E-cad^ or H^inv^/L^E-cad^ OSCCs, when plated on laminin, showed large and elongated adhesions probably due to the epithelial origin of the tumor. However, specifically in H^inv^/L^E-cad^ tumor cells, fibronectin induced smaller cell-ECM adhesions with a fast turnover, which reflected in increased Rac1 activation [46] and faster single cell migration. Thus the ability to metastasize from a laminin to a fibronectin environment might reflect a switch from cadherin-mediated cell-cell adhesions to signaling integrin-ECM adhesions, which promote directional cell migration.

**Fig 6:**
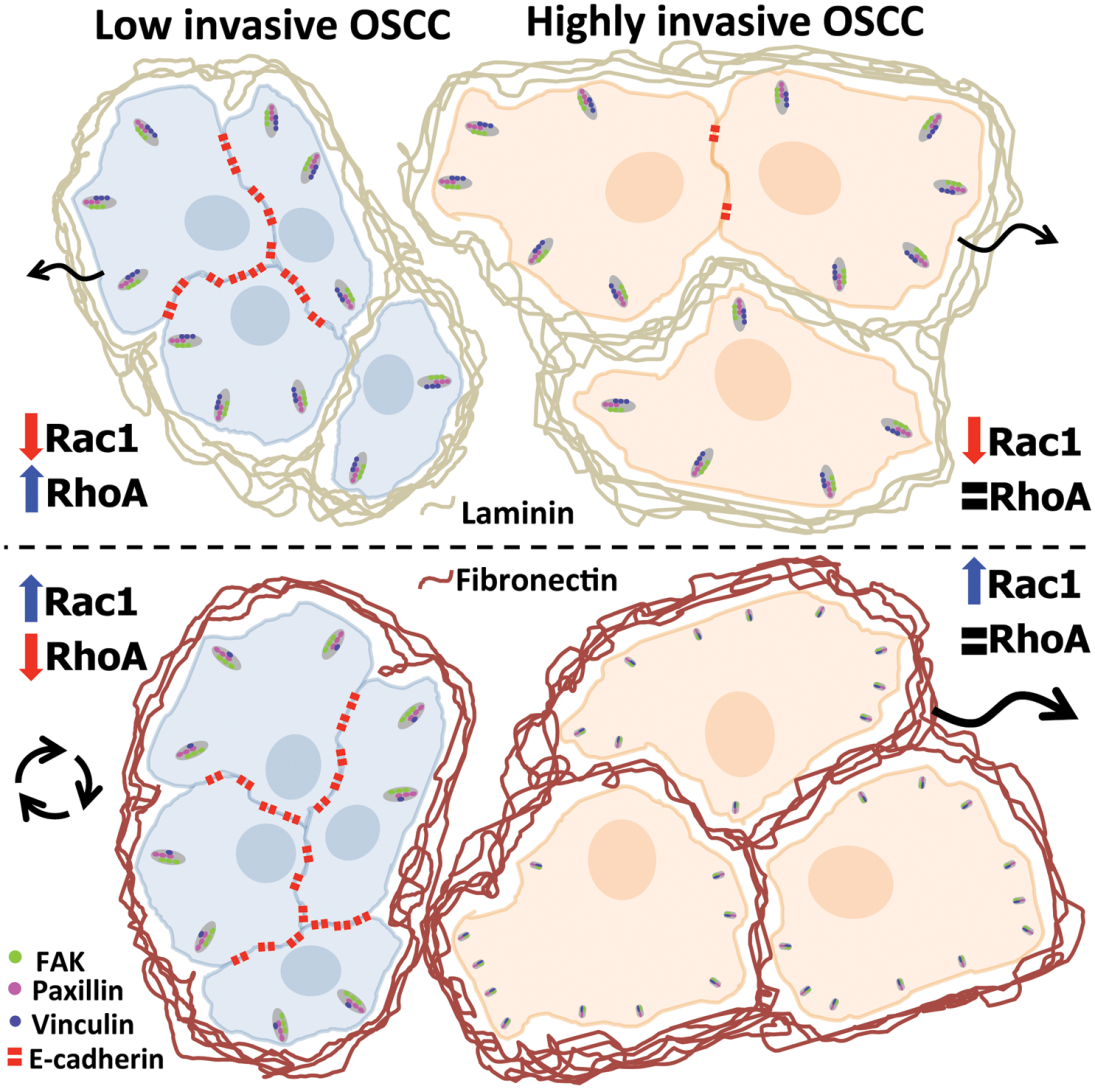
Effects of the differential composition of extracellular matrix on cell adhesion and signaling of Oral Squamous Cell Carcinoma. OSCC with high E-cadherin levels (blue cells) shows collective and single cell migration in the presence of laminin and collective non-directional migration in fibronectin. This switch correlated to an increase in Rac1 and a decrease on RhoA activation and modulation of the vinculin levels in adhesion, induced by the fibronectin-enriched environment. For OSCCs with low E-cadherin levels (orange cells), fibronectin induced smaller adhesions and increased Rac1 signaling, which correspond to a fast single cell migration phenotype. This model proposes that the ECM composition can trigger the tumor invasive behavior according to differentiation levels of OSCC cells.

Several reports associate differential activation of adhesion-related proteins with a worse patient prognosis [47–49]. Also, vulvar squamous cell carcinoma tumors silenced for the fibronectin binding protein, integrin β1, show a more encapsulated and less invasive profile [50] indicating that cell-ECM interaction is an important player during tumorigenesis. Consistent with these findings, we demonstrated that human OSCC biopsies show decreased junctional E-cadherin levels at the center of the tumor when compared to the epithelia adjacent to the tumor, while the cell-ECM adhesion proteins paxillin, vinculin and FAK showed a differential distribution in cancer cells at the border of tumor islands close to regions of contact to the fibronectin enriched ECM. Therefore, our results (Fig 6) show that the extracellular matrix composition is able to influence the pattern of tumor invasion and metastasis according to the differentiation level of the tumor cells, probably through modulation of cell signaling and changes in the balance between cell-cell and cell-ECM adhesion. These data suggest that the invasive behavior of OSCC not only relies on intrinsic factors (i.e. mutations and abnormal expression of proteins) but also on extrinsic factors (such as the ECM composition), which could help to understand the failure of some tumor therapies and contribute to development of new anti-tumorigenic approaches.

## Supporting Information

S1 Fig Differential distribution of E-cadherin on two different cell lines. L^inv^/H^E-cad^ (A) or H^inv^/L^E-cad^ (B) OSCC were plated on laminin or fibronectin, fixed and stained for E-cadherin and actin. Scale bar = 20μm.
(TIF)

S2 Fig Distribution of cell-cell and cell-ECM adhesion molecules in OSCC human biopsies. Original images showed in Fig 5 in the manuscript. Biopsies corresponding to the epithelia adjacent to the tumor (A) and from the center of the tumor region (B) were submitted to actin staining (first column) and immunostaining (second column) for E-cadherin, paxillin, vinculin or FAK. Representative images of n = 10, digital zoom 5x, scale bar = 20μm.
(TIF)

S3 Fig Distribution of fibronectin in OSCC human biopsies. Regions of biopsies corresponding to the epithelia adjacent to the tumor (A) and from the center of the tumor (B) were submitted to immunostaining for fibronectin (green) and actin staining (magenta). Representative images from the same patient (n = 10), scale bar = 50μm.
(TIF)

S1 Movie Migratory properties of low and highly invasive Oral Squamous Cell Carcinoma cell lines plated on Matrigel. Time-lapse images (right column) and cell tracking (left column) of Oral Squamous Cell Carcinoma with H^inv^/L^E-cad^ (upper line) or L^inv^/H^E-cad^ (lower line) plated for 1h on Matrigel (50μl/cm^2^) and imaged for 24h with a 10min time interval.
(AVI)

S2 Movie Migratory properties of low invasive Oral Squamous Cell Carcinoma plated in laminin or fibronectin. Time-lapse images (left column) and cell tracking (right column) of Oral Squamous Cell Carcinoma with L^inv^/H^E-cad^ plated for 1h on laminin (2μg/ml, upper line) or fibronectin (2μg/ml, lower line) and imaged for 24h with a 10min time interval. This movie corresponds to Fig 1B and 1C.
(AVI)

S3 Movie Migratory properties of highly invasive Oral Squamous Cell Carcinoma plated in laminin or fibronectin. Time-lapse images (left column) and cell tracking (right column) of H^inv^/L^E-cad^ plated for 1h on laminin (2μg/ml, upper line) or fibronectin (2μg/ml, lower line) and imaged for 24h with a 10min time interval. This movie corresponds to Fig 1D and 1E.
(AVI)

S4 Movie Migratory properties of low invasive Oral Squamous Cell Carcinoma plated in a 3D matrix. Time-lapse images (left column) and cell tracking (right column) of L^inv^/H^E-cad^ OSCC were plated for 1h in a 3D matrix of collagen (1.2mg/ml, upper line), collagen+laminin (1.2mg/ml+10μg/ml, center line) or collagen+fibronectin (1.2mg/ml+10μg/ml, lower line) and imaged for 24h with a 10min time interval. This movie corresponds to Fig 1F.
(AVI)

S5 Movie Migratory properties of highly invasive Oral Squamous Cell Carcinoma plated in a 3D matrix. Time-lapse images (left column) and cell tracking (right column) of H^inv^/L^E-cad^ OSCC were plated for 1h in a 3D matrix of collagen (1.2mg/ml, upper line), collagen+laminin (1.2mg/ml+10μg/ml, center line) or collagen+fibronectin (1.2mg/ml+10μg/ml, lower line) and imaged for 24h with a 10min time interval. This movie corresponds to Fig 1F.
(AVI)

S6 Movie Migratory properties of Oral Squamous Cell Carcinoma cell lines-derived spheroids in a 3D extracellular matrix. Time-lapse images of spheroids obtained from Oral Squamous Cell Carcinoma with L^inv^/H^E-cad^ (left column) plated in a 3D matrix containing collagen+fibronectin (1.2mg/ml+10μg/ml) or H^inv^/L^E-cad^ (center and right column) plated in a 3D extracellular matrix composed by collagen only (1.2mg/ml, center column) or collagen+fibronectin (1.2mg/ml+10μg/ml, right column) and imaged for 36h with a 10min time interval.
(AVI)

S7 Movie Extracellular matrix composition affects adhesion dynamics of highly invasive Oral Squamous Cell Carcinoma. Adhesion dynamics of Oral Squamous Cell Carcinoma with low E-cadherin levels transfected with paxillin-GFP, plated for 20min on laminin (2μg/ml, left column) or fibronectin (2μg/ml, right column) and imaged using Total Internal Reflectance Fluorescent (TIRF) microscopy for 10min with a 3s time interval. The black box represents a digital zoom of the original movie showing the details of cell adhesion dynamics in each condition.
(AVI)
