# Supplementary figures and images for: Fibronectin Modulates Cell Adhesion and Signaling to Promote Single Cell Migration of Highly Invasive Oral Squamous Cell Carcinoma
Source: PLoS One. 2016 Mar 15;11(3):e0151338. doi: 10.1371/journal.pone.0151338 (PMC4792484; doi:10.1371/journal.pone.0151338)

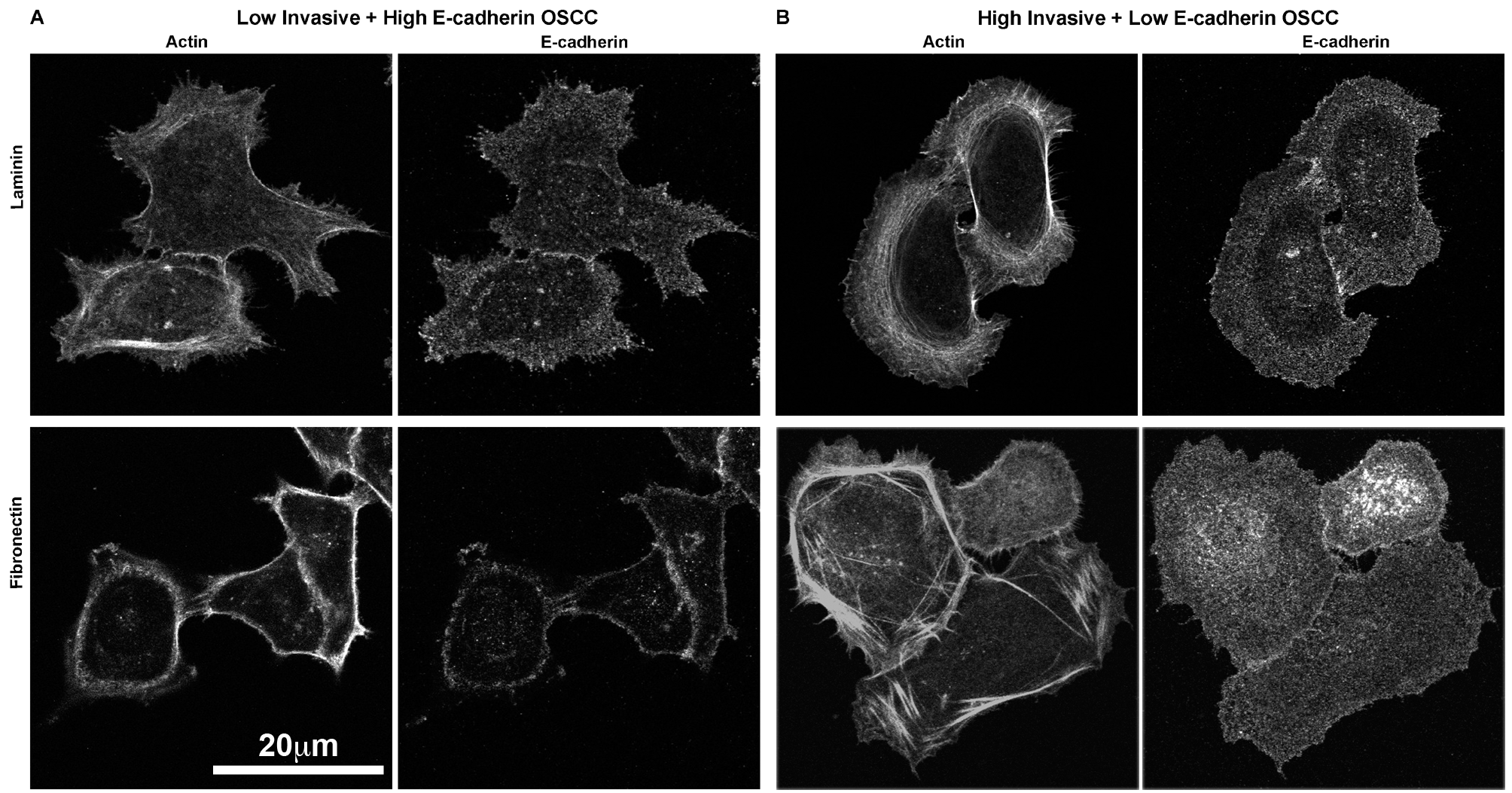

Supplement: S1 Fig — Linv/HE-cad (A) or Hinv/LE-cad (B) OSCC were plated on laminin or fibronectin, fixed and stained for E-cadherin and actin. Scale bar = 20μm. (TIF) [file pone.0151338.s001.tif]

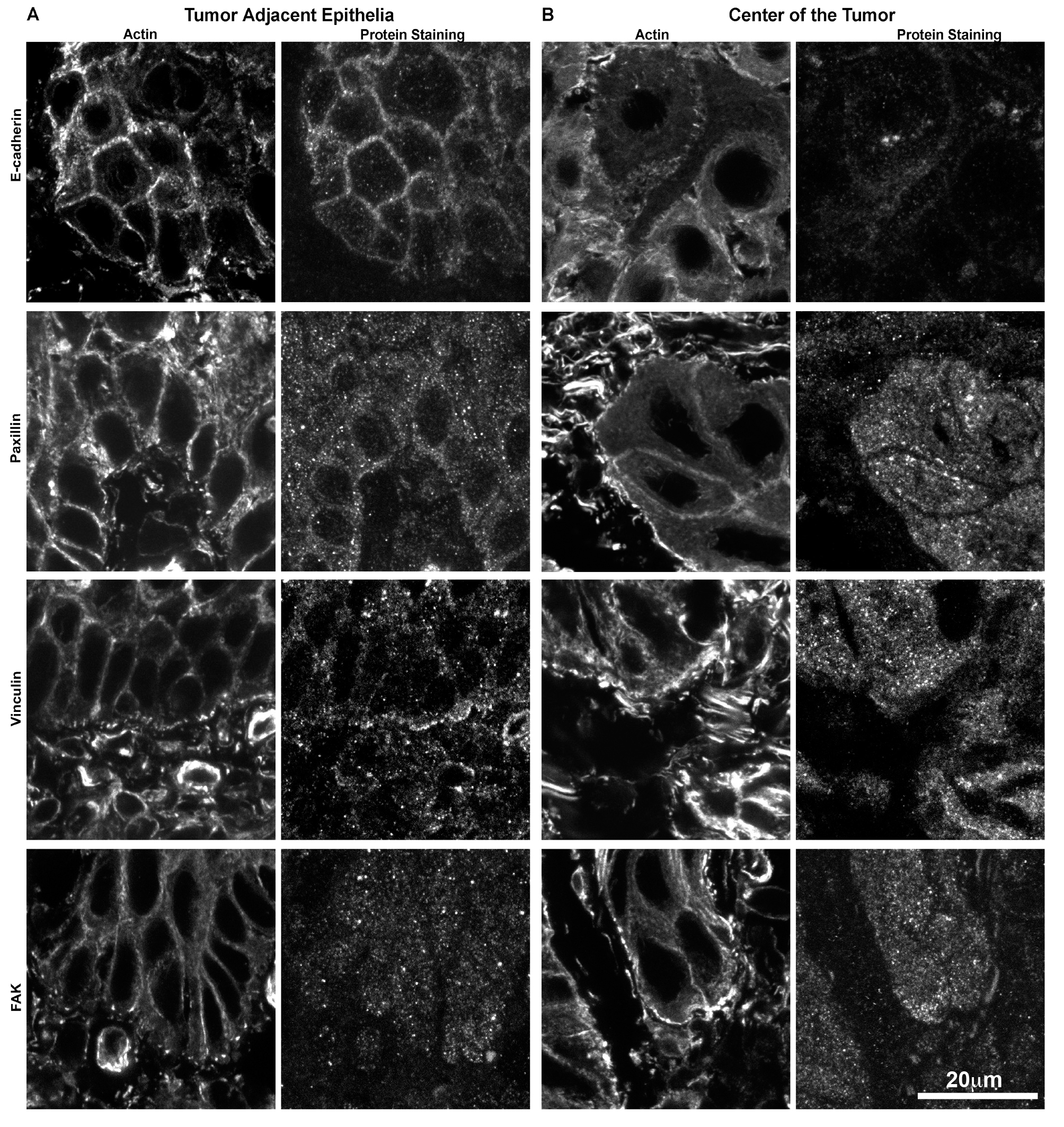

Supplement: S2 Fig — Original images showed in Fig 5 in the manuscript. Biopsies corresponding to the epithelia adjacent to the tumor (A) and from the center of the tumor region (B) were submitted to actin staining (first column) and immunostaining (second column) for E-cadherin, paxillin, vinculin or FAK. Representative images of n = 10, digital zoom 5x, scale bar = 20μm. (TIF) [file pone.0151338.s002.tif]

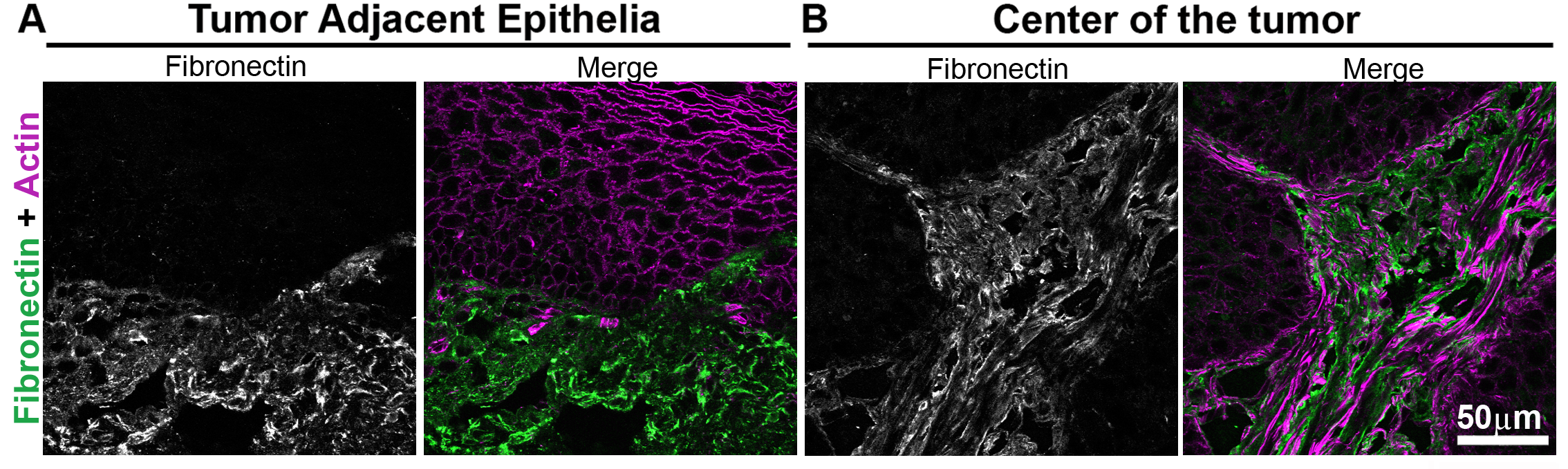

Supplement: S3 Fig — Regions of biopsies corresponding to the epithelia adjacent to the tumor (A) and from the center of the tumor (B) were submitted to immunostaining for fibronectin (green) and actin staining (magenta). Representative images from the same patient (n = 10), scale bar = 50μm. (TIF) [file pone.0151338.s003.tif]
